# Expansion of myeloid-derived suppressor cells with arginase activity lasts longer in aged than in young mice after CpG-ODN plus IFA treatment

**DOI:** 10.18632/oncotarget.3626

**Published:** 2015-04-13

**Authors:** María F. Harman, Romina P. Ranocchia, Carolina V. Gorlino, María F. Sánchez Vallecillo, Sofía D. Castell, María I. Crespo, Belkys A. Maletto, Gabriel Morón, María C. Pistoresi-Palencia

**Affiliations:** ^1^ Centro de Investigación en Bioquímica Clínica e Inmunología, Consejo Nacional de Investigaciones Científicas y Técnicas, Facultad de Ciencias Químicas, Universidad Nacional de Córdoba, Córdoba, Argentina

**Keywords:** aging, myeloid-derived suppressor cells, arginase, CpG-ODN, immunomodulation

## Abstract

As we age, the homeostatic function of many systems in the body, such as the immune function declines, which in turn contributes to augment susceptibility to disease. Here we describe that challenging aged mice with synthetic oligodeoxynucleotides containing unmethylated cytosine guanine motifs (CpG-ODN) emulsified in incomplete Freund's adjuvant (IFA), (CpG-ODN+IFA) an inflammatory stimulus, led to the expansion of CD11b^+^Gr1^+^ myeloid cells with augmented expression of CD124 and CD31. These myeloid cells lasted longer in the spleen of aged mice than in their younger counterparts after CpG-ODN+IFA treatment and were capable of suppressing T cell proliferative response by arginase induction. Myeloid cells from aged CpG-ODN+IFA-treated mice presented increased arginase-1 expression and enzyme activity. In addition, we found a different requirement of cytokines for arginase induction according to mice age. In myeloid cells from young treated mice, arginase-1 expression and activity is induced by the presence of each IL-4 or IL-6 in their extracellular medium, unlike myeloid cells from aged treated mice which need the presence of both IL-4 and IL-6 together for arginase induction and suppressor function.

## INTRODUCTION

Myeloid-derived suppressor cells (MDSCs) are a heterogeneous population of cells that consist of myeloid progenitors and immature myeloid cells [1]. Immature myeloid cells with the same phenotype as MDSCs are continually generated in the bone marrow of healthy individuals and differentiate into mature myeloid cells: granulocytes, macrophages or dendritic cells [1–2]. However, under inflammatory conditions, MDSC levels are elevated in peripheral secondary lymph organs in both human [3] and murine [4–6] hosts. MDSCs regulate both innate and adaptative immunity [7], and are responsible for suppressing T-cell responses [1]. MDSCs are identified in mice by the co-expression of the myeloid-cell lineage differentiation antigen CD11b and Gr1 [1]. A number of cytokines and transcription factors were shown to modulate the expansion and activation of MDSCs [1, 8]. MDSC exerts its suppressive activity upregulating the expression of factors such as arginase-1 and iNOS, as well as increasing the production of nitric oxide and reactive oxygen species [1].

Aging impacts the homeostatic function of many systems in the body, including the immune system, which reduces the capacity to mount a robust immune response [9–11]. As a result, elderly individuals experience a higher incidence of various diseases, such as autoimmune disorders, infections and cancer.

We have previously demonstrated that CpG-ODN used as an adjuvant was able to induce a specific immune response in aged mice comparable to that in young mice [12–14]. We have also demonstrated that systemic administration of CpG-ODN emulsified in incomplete Freund's adjuvant (CpG-ODN+IFA) induced transitory expansion of splenic MDSCs with CD11b^+^Gr1^+^ phenotype in young mice, which caused suppression of T-cell proliferation mediated by arginase activity [15]. Thus, CpG-ODN, besides being a powerful immunostimulatory compound, is capable of inducing MDSCs as a counter-regulatory response in young mice.

Based on all these considerations, we aimed to investigate the effects of aging on the expansion and suppressor function of myeloid cells in CpG-ODN+IFA-treated mice.

The data presented in this work show that CpG-ODN+IFA is capable of inducing the expansion of myeloid cells in aged mice, which remain elevated for a longer time than in their younger counterparts. Myeloid cells from aged treated mice suppress the T cell proliferative response by arginase induction. Here we found a different requirement of cytokines for arginase induction according to mice age. In myeloid cells from young treated mice, arginase-1 expression and activity is induced by the presence of each IL-4 or IL-6 in their extracellular medium, unlike myeloid cells from aged treated mice which need the presence of both IL-4 and IL-6 together for arginase induction and suppressor function. Further investigation of the functional consequences of elevated MDSCs for more time facing an inflammatory stimulus will provide valuable insight into the progression of age-related pathologies.

## RESULTS

### CD11b^+^Gr1^+^ myeloid cells accumulate in the spleen of CpG-ODN+IFA-treated aged mice

**Figure 1:**
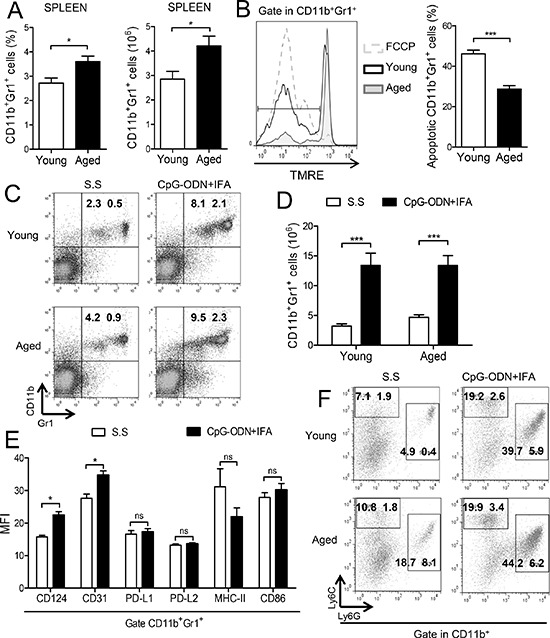
Myeloid cells accumulate in spleen of CpG-ODN+IFA-treated aged mice (A–B) Splenocytes from young and aged mice were stained with anti-CD11b and anti-Gr1 antibodies and analyzed by flow cytometry. **(A)** Percentage and absolute number of CD11b^+^Gr1^+^ cells are presented. **(B)** Percentage of apoptotic myeloid cells determined by TMRE staining (TMRE^low^ cells) on CD11b^+^Gr1^+^ gated splenocytes from young and aged mice, after 18 h of culture without stimuli. Cells incubated with FCCP were used as positive control. (C–E) Splenocytes were collected from mice ten days after CpG-ODN+IFA or Saline solution (S.S) treatment and analyzed by flow cytometry. **(C)** Representative dot plots with percentages (***p < 0.001 young CpG-ODN+IFA vs S.S, **p < 0.01 aged CpG-ODN+IFA vs S.S; mean ± SEM) and **(D)** absolute number of CD11b^+^Gr1^+^ cells in spleen from young and aged mice are presented. **(E)** Mean Fluorescence Intensity (MFI) for the indicated molecules on CD11b^+^Gr1^+^ gated cells from spleen of aged mice after CpG-ODN+IFA or S.S treatment. **(F)** Representative dot plots and percentages of CD11b^+^Ly6G^−^Ly6C^high^ and CD11b^+^Ly6G^+^Ly6C^low^ cells are shown as mean ± SEM; granulocytic population: **p < 0.01 CpG-ODN+IFA vs S.S (young and aged), monocytic population: **p < 0.01 CpG-ODN+IFA vs S.S (young and aged). Data are from (A, C–D) four and (B, E–F) three independent experiments; mean ± SEM (*n* = 4 mice/group) *p < 0.05; **p < 0.01; ***p < 0.001.

It is well known that human and murine hematopoietic stem cells exhibit cell autonomous changes during aging, showing attenuated lymphoid lineage output, while myeloid lineage potential is maintained or even increased [10–11, 16]. For this reason, we first evaluated CD11b^+^Gr1^+^ myeloid cells in aged mice. Spleen, blood and bone marrow compartments of aged mice contained higher numbers of CD11b^+^Gr1^+^ cells compared to young animals (Figure 1A and Supplementary Figure 1A). Similar results have been recently reported by other authors [17–18] although we further observed a lower percentage of apoptotic CD11b^+^Gr1^+^ cells from aged mice compared to young animals indicating that they have a prolonged life span (Figure 1B), which may be another reason for their increased number in aged mice.

We have recently reported that the numbers of CD11b^+^Gr1^+^ cells were increased in the spleen of young BALB/c mice after a single administration of CpG-ODN+IFA [15]. With this in mind, we investigated whether CpG-ODN+IFA could induce CD11b^+^Gr1^+^ cells expansion in aged mice. As shown in Figure 1C and 1D, 10 days after CpG-ODN+IFA-treatment, the percentage and absolute number of CD11b^+^Gr1^+^ cells were significantly augmented in spleen of aged mice compared to saline solution-treated mice. Although the expansion of myeloid cells after treatment reached similar levels as in their younger counterparts their induction was lower because of their basal augmented number (Supplementary Figure 1B).

In order to evaluate the expression of myeloid lineage differentiation and maturation markers in myeloid cells that accumulated in the spleen of aged mice after CpG-ODN+IFA treatment, flow cytometry analysis was performed. We observed upregulated expression of CD124 (IL-4Rα) and CD31; however, no significant differences were found in the expression of PD-L1, PD-L2, MHC-II and CD86 in these cells (Figure 1E).

Recent reports indicated that MDSCs can be divided into two distinct subsets based on their expression of two Gr1 epitopes, Ly6G and Ly6C: granulocytic MDSCs with CD11b^+^Ly6G^+^Ly6C^low^ phenotype and monocytic MDSCs with CD11b^+^Ly6G^−^Ly6C^high^ phenotype [1, 6, 19]. After CpG-ODN+IFA treatment, both monocytic and granulocytic subpopulations were increased in spleen of aged and young mice (Figure 1F); however, the granulocytic subset was the predominant population of myeloid cells that expanded (Figure 1F). As spleens of aged saline solution-treated mice harbor higher numbers of myeloid cells the increase of both subsets after treatment was lower in these animals than in their younger counterparts.

Collectively our data indicate that secondary lymphoid organs of aged mice harbor an elevated number of CD11b+Gr1+ myeloid cells which are less sensitive to spontaneous apoptosis than their younger counterparts. Besides, after CpG-ODN+IFA-treatment of aged mice, this myeloid cell population expanded and presented phenotype characteristics of MDSCs.

### Myeloid cells from aged CpG-ODN+IFA-treated mice suppress T cell proliferative response

**Figure 2:**
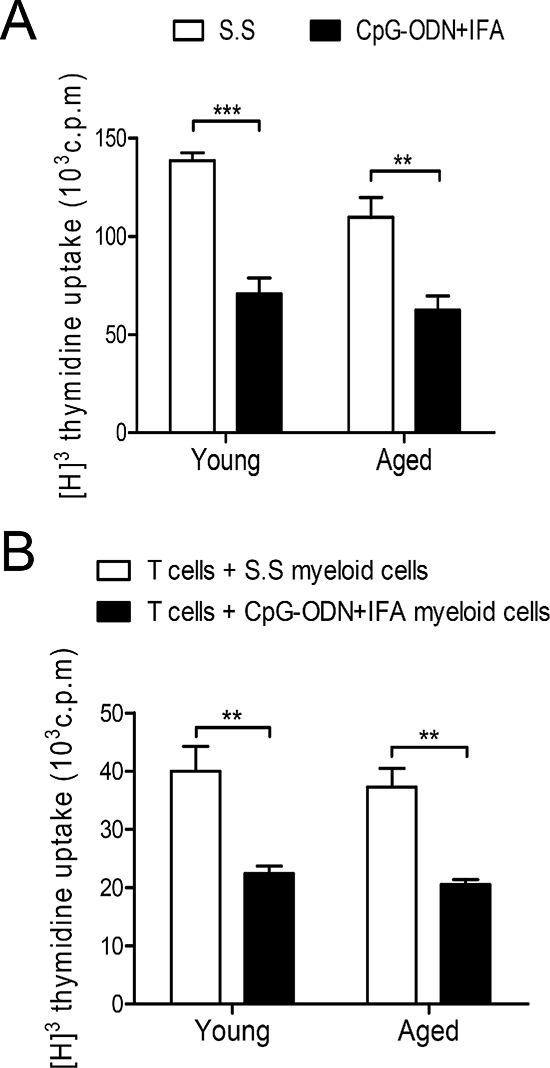
Myeloid cells from aged CpG-ODN+IFA-treated mice suppress T cell proliferation Spleens were collected from young and aged mice after ten days of CpG-ODN+IFA-treatment. **(A)** Splenocytes were stimulated with ConA or RPMI (unstimulated) and cultured for 72 h. **(B)** Naïve T cells isolated from young mice were stimulated with anti-CD3 plus anti-CD28 and co-cultured, at a 1:1 ratio, with myeloid cells purified from young or aged S.S or CpG-ODN+IFA-treated mice for 48 h. (A–B) T cell proliferation was measured by [^3^H]-Thymidine incorporation after 18 h pulse. Values are represented as c.p.m of stimulated minus unstimulated. (A–B) Data are representative of three independent experiments; mean ± SEM (*n* = 4 mice/group) **p < 0.01; ***p < 0.001.

MDSCs which accumulate during cancer, inflammation and infection have a remarkable ability to suppress T cell responses, and this function is their defining characteristic [1]. First, we performed an *in vitro* proliferative assay of splenocytes to evaluate the effect of the expansion of myeloid cells by CpG-ODN+IFA treatment. We observed a reduction in the proliferative response to ConA of splenocytes from aged mice after CpG-ODN+IFA treatment, similar to that occurring in splenocytes from young treated mice (Figure 2A). To examine if the lower proliferative response was due to the expansion of the myeloid cell population with suppressor function, we evaluated the suppressor activity of myeloid cells isolated from spleen of aged CpG-ODN+IFA-treated mice. T-cells from young syngeneic mice stimulated with anti-CD3 plus anti-CD28 were used as responders. T cell proliferative response was lower when they were cultured with myeloid cells from aged CpG-ODN+IFA-treated mice, compared to cultures with myeloid cells from saline solution-treated aged mice (Figure 2B). Interestingly the reduction of T cell proliferation was similar when the co-cultures were performed with myeloid cells isolated from young or aged treated mice.

The results indicate that myeloid cells from aged CpG-ODN+IFA-treated mice are capable of suppressing T-cell proliferative response as effectively as myeloid cells from young treated mice.

### Myeloid cells from aged CpG-ODN+IFA-treated mice suppress T cell proliferation by arginase

**Figure 3:**
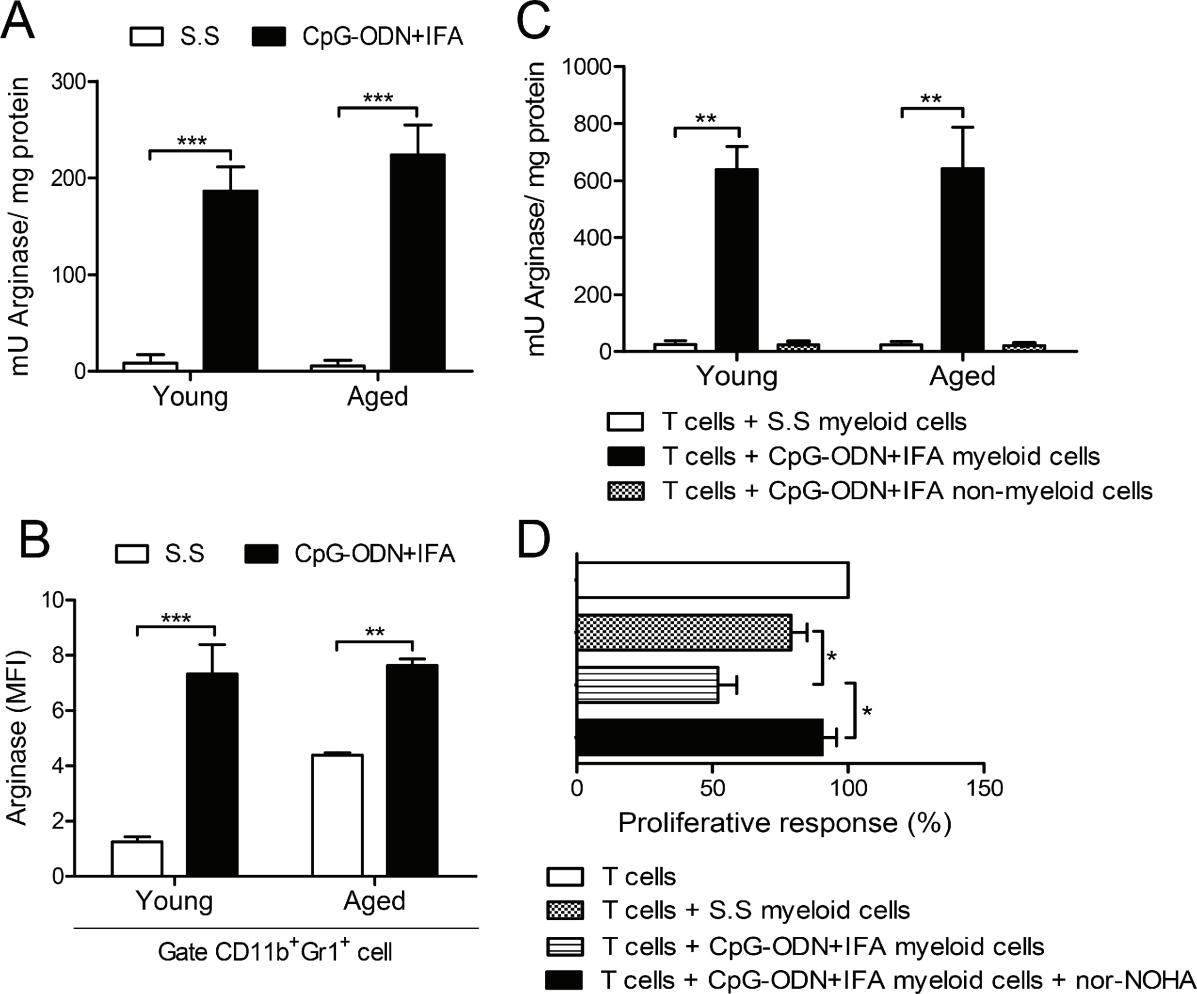
Myeloid cells from aged CpG-ODN+IFA-treated mice suppress T cell proliferation by arginase Spleens were obtained from mice after ten days of CpG-ODN+IFA or S.S-treatment. **(A)** Splenocytes from young and aged mice were stimulated with ConA or RPMI for 72 h, lysed and prepared for arginase activity analysis. **(B)** Mean Fluorescence Intensity (MFI) for arginase-1 on CD11b^+^Gr1^+^ gated splenocytes from young and aged mice after 48 h culture with ConA or RPMI. **(C)** Isolated myeloid cells from young and aged S.S or CpG-ODN+IFA-treated mice were co-cultured with naïve T-cells isolated from young mice stimulated with anti-CD3 plus anti-CD28 and then prepared for arginase activity analysis. CD11b^−^ spleen cells from CpG-ODN+IFA-treated-mice (non-myeloid cells) were used as control. (A and C) Results are expressed as mU of enzyme activity per mg of protein lysate (Stimulated minus unstimulated). **(D)** Isolated naïve T-cells from young mice were stimulated with anti-CD3 plus anti-CD28 and co-cultured with purified myeloid cells, from aged S.S or CpG-ODN+IFA-treated mice, in the presence or absence of nor-NOHA, T-cell proliferation was measured by [^3^H]-Thymidine incorporation after 18 h pulse, values are represented as fold increase related to stimulated T-cell alone proliferative response (100%). Results are representative of (A and C) three and (B and D) two experiments performed (mean ± SEM *n* = 4 mice/group) *p < 0.05; **p < 0.01; ***p < 0.001.

We have previously shown that the T cell suppressor ability of myeloid cells from young mice after CpG-ODN+IFA treatment was linked to a mechanism based on L-arginine depletion by arginase activity [15]. We therefore investigated whether arginase activity was induced in splenocytes of aged mice after CpG-ODN+IFA treatment. As shown in Figure 3A, splenocytes from aged treated mice exhibited greater arginase activity than splenocytes from their saline solution-treated counterparts. Intracellular staining showed increased arginase-1 protein expression in CD11b^+^Gr1^+^ cells from aged and young mice after CpG-ODN+IFA treatment (Figure 3B). To confirm these results, myeloid cells from aged CpG-ODN+IFA-treated mice were isolated and cultured with stimulated T cells from young mice. Arginase activity increased in these myeloid cells and, as expected, no activity was detected in the negative fraction (Figure 3C). Similar results were obtained in cultures of myeloid cells from young CpG-ODN+IFA-treated mice (Figure 3C). Interestingly, myeloid cells from aged saline solution-treated mice showed higher arginase-1 expression compared to their younger counterparts (Figure 3B) although no arginase activity was observed in these cells (Figure 3C).

Our results suggest that there is a close correlation between arginase activity in myeloid cells from aged CpG-ODN+IFA-treated mice and their capacity to regulate T-cell proliferation. To examine this issue, the arginase inhibitor, nor-NOHA, was added to the co-cultures of stimulated T-cells and myeloid cells isolated from aged CpG-ODN+IFA-treated mice. As shown in Figure 3D, T cell proliferative response was restored by nor-NOHA, showing similar proliferation levels to that of T-cells cultured with myeloid cells from saline solution-treated mice or T cells alone.

These findings demonstrate that the induction of arginase is one of the major mechanisms involved in the suppressive capacity of myeloid cells from aged CpG-ODN+IFA-treated mice.

### Myeloid-derived suppressor cell expansion lasts longer in aged than in young mice after CpG-ODN+IFA treatment

**Figure 4:**
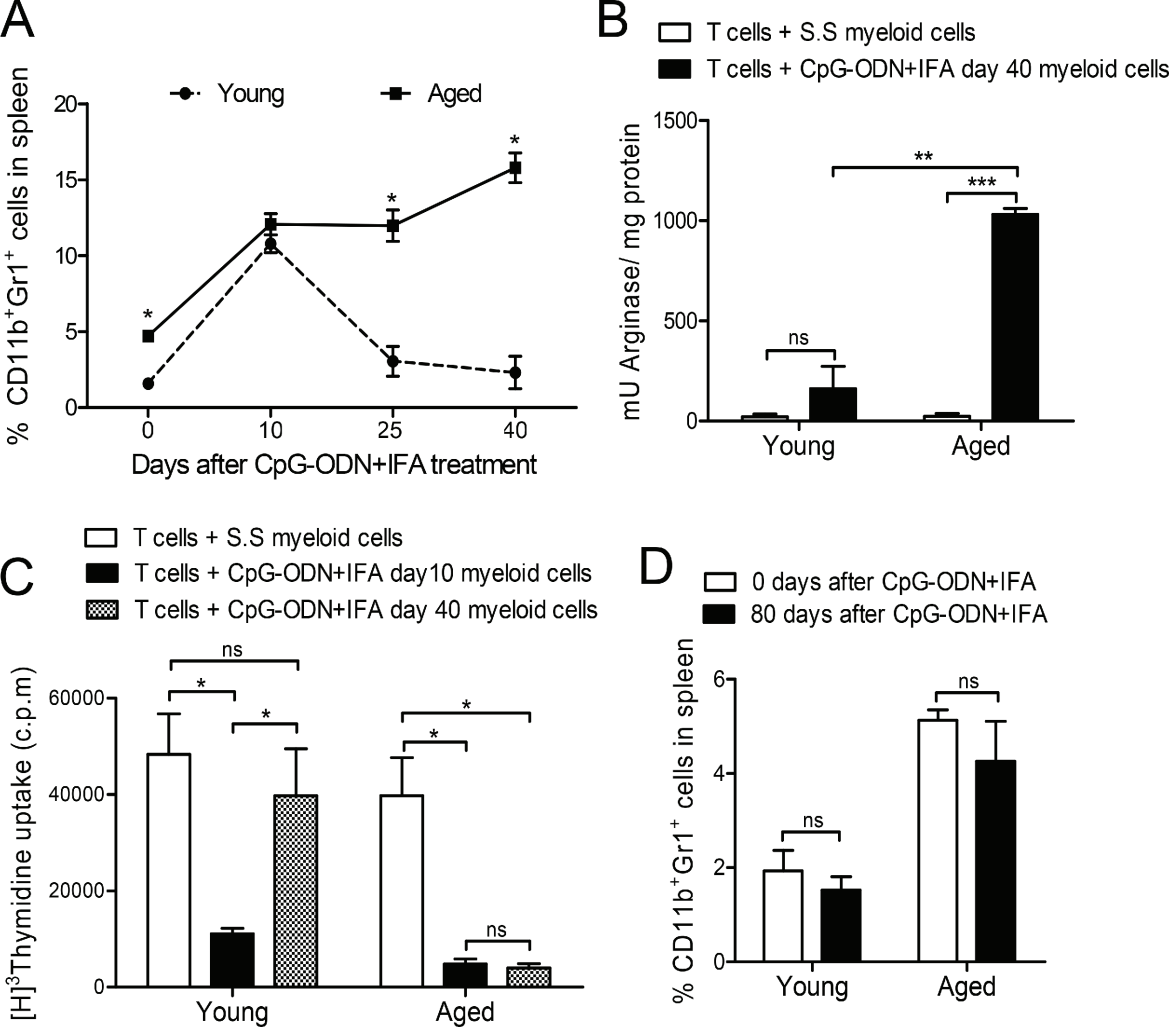
Myeloid suppressor cell expansion lasts longer in aged than in young mice after CpG-ODN+IFA treatment **(A)** Splenocytes from young and aged mice were obtained 0, 10, 25 and 40 days after treatment, stained with anti-CD11b and anti-Gr1 and analyzed by flow cytometry. **(B)** Myeloid cells were isolated from young and aged mice 40 days after S.S or CpG-ODN+IFA- treatment and cultured with naïve T cells from young mice stimulated with anti-CD3 and anti-CD28. Cells were prepared for arginase activity analysis. Results are expressed as mU of enzyme activity per mg of protein lysate. **(C)** Ten and 40 days after treatment, myeloid cells were isolated from spleen of young and aged treated mice and cultured with naïve T-cells from young mice stimulated with anti-CD3 and anti-CD28. T-cell proliferation was measured by [^3^H]-Thymidine incorporation after 18 h pulse, (values are represented as c.p.m of stimulated minus unstimulated). **(D)** Zero or 80 days after treatment, splenocytes were removed from mice, stained with anti-CD11b and anti-Gr1 and analyzed by flow cytometry. (A–D) Results are representative of three experiments performed (mean ± SEM; *n* = 4 mice/group). *p < 0.05; **p < 0.01; ***p < 0.001.

We next asked how long it takes for myeloid cells to return to basal levels in aged mice after CpG-ODN+IFA treatment. We studied these cells at different time points after treatment. As mentioned before, 10 days after treatment there was a marked expansion of myeloid cell population in young mice and as shown in Figure 4A, they returned to basal levels on day 25. In contrast, the population of myeloid cells that expanded on day 10 in aged mice remained elevated at least 40 days after CpG-ODN+IFA-treatment (Figure 4A).

We next isolated myeloid cells from mice 40 days after CpG-ODN+IFA-treatment and cultured them with stimulated T cells in order to evaluate the preservation of their immunosuppressive capacity. At this time point, myeloid cells obtained from aged mice conserved high arginase activity whereas myeloid cells from young animals presented the same levels as their saline solution counterparts (Figure 4B). A similar result was observed with total splenocytes (data not shown). Moreover, T cell proliferative response was reduced when myeloid cells isolated from aged mice 40 days after CpG-ODN+IFA-treatment were added to the co-cultures, indicating that these cells were still suppressive (Figure 4C). In contrast, myeloid cells purified from young mice after 40 days of treatment did not suppress T cell proliferation (Figure 4C).

Finally, myeloid cell levels in spleen of aged mice 80 days after CpG-ODN+IFA-treatment were the same as those observed in their saline solution counterparts (Figure 4D), indicating that the expanded population had already returned to its basal levels by this time.

### Environmental IL-4 and IL-6 are involved in arginase induction in myeloid cells from CpG-ODN+IFA-treated mice

**Figure 5:**
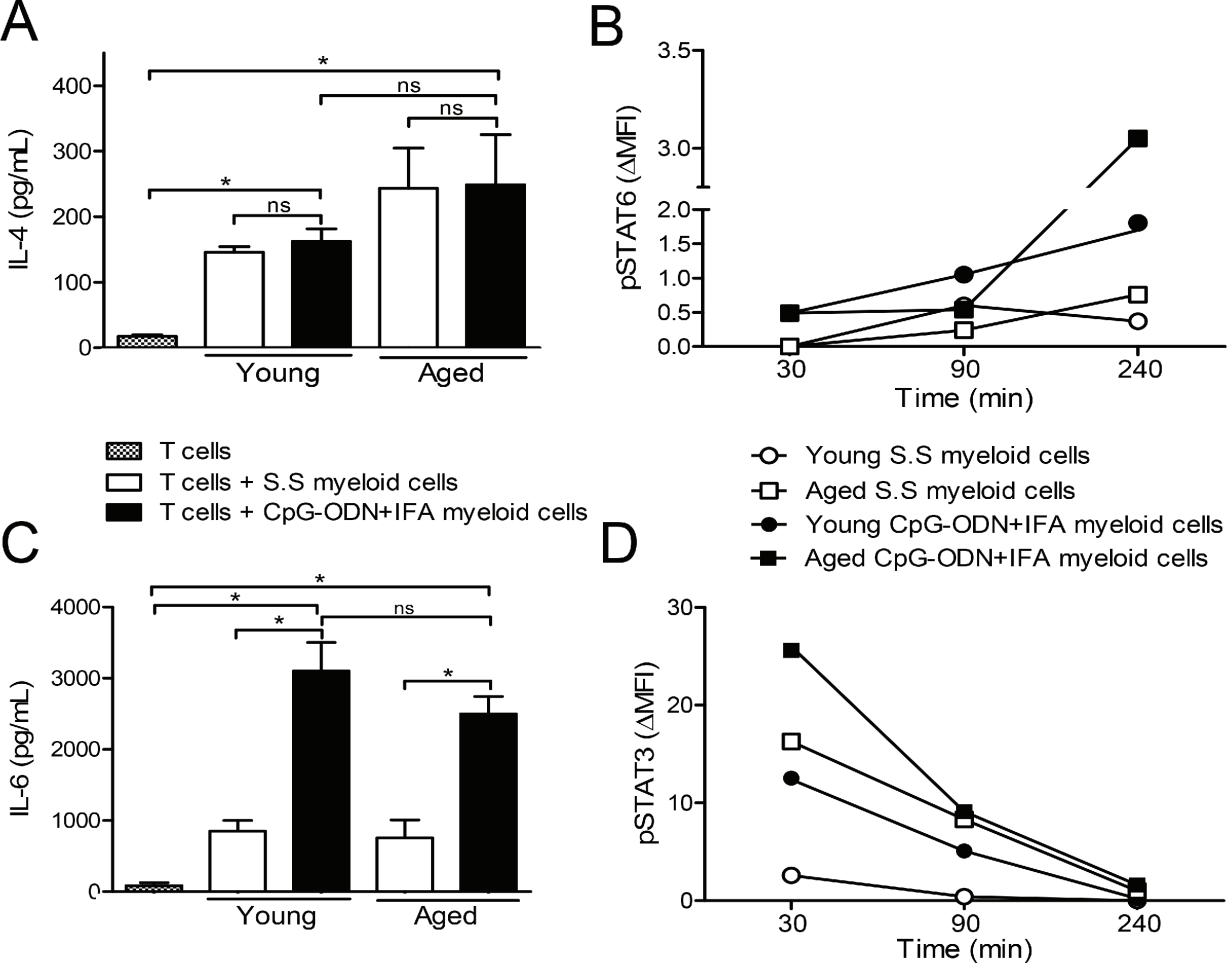
IL-4 and IL-6 in the extracellular medium of myeloid cells from CpG-ODN+IFA-treated mice After 10 days of S.S or CpG-ODN+IFA-treatment, myeloid cells were isolated from the spleen of young and aged mice and cultured with naïve T cells isolated from young mice stimulated with anti-CD3 plus anti-CD28. (A and C) After 48 h of co-culture, supernatants were collected. **(A)** IL-4 and **(C)** IL-6 levels were measured by ELISA. (B and D) After 30, 90 and 240 min of co-culture, cells were collected, fixed, permeabilized, and stained with anti-CD11b, anti-Gr1, anti-STAT6(pY641) and anti-STAT3(pY705). **(B)** pSTAT6 and **(D)** pSTAT3 levels were analyzed on gated CD11b^+^Gr1^+^ myeloid cells. Results represent the expression (MFI) of pSTAT3 and pSTAT6 at 30, 90 and 240 min by subtracting that measured at zero time (ΔMFI). Data are shown from one representative experiment of (A–C) three, (B–D) two performed (mean ± SEM *n* = 4 mice/group) *p < 0.05.

Myeloid cells can be activated in response to local environmental signals. It has been established that IL-4 activating STAT6 may be a pathway that induces the expression of arginase-1 in MDSCs [1]. We found that IL-4 levels were augmented when stimulated T cells were co-cultured with myeloid cells from young or aged saline solution or CpG-ODN+IFA-treated mice (Figure 5A). In addition, although no significant differences were observed in IL-4 levels between the co-cultures of myeloid cells from saline solution and CpG-ODN+IFA-treated mice (Figure 5A), we found that, after 240 min of co-culture, myeloid cells from young and aged CpG-ODN+IFA-treated mice presented increased STAT6 phosphorylation (Figure 5B). In line with these data, we found that CD124 (IL-4Rα) expression was upregulated in myeloid cells from CpG-ODN+IFA-treated mice at both ages (Figure 1E and Supplementary Figure 2), which may be promoting IL-4 dependent activation of STAT6 in these cells.

In addition, recent studies showed that, among others, IL-6 was responsible for the phosphorylation of STAT3 and the expression of arginase-1 in myeloid cells [20–22]. We observed increased IL-6 levels in co-cultures of stimulated T cells with myeloid cells from young or aged CpG-ODN+IFA-treated mice compared to their saline solution counterparts (Figure 5C). Consistent with these results, after 30 min of co-culture, myeloid cells from young and aged CpG-ODN+IFA-treated mice presented increased STAT3 phosphorylation (Figure 5D). These results correlated with the induced arginase activity and expression in myeloid cells from CpG-ODN+IFA-treated mice (Figure 3B and 3C).

**Figure 6:**
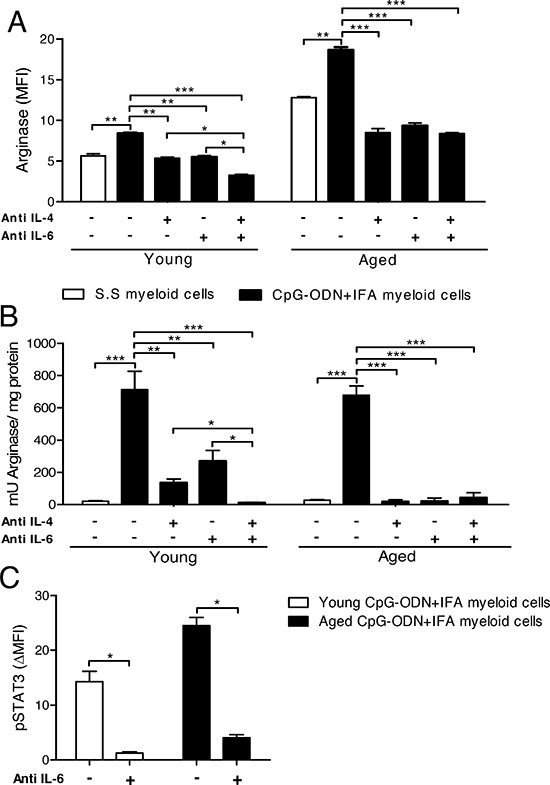
Environmental IL-4 and IL-6 are involved in arginase induction in myeloid cells from CpG-ODN+IFA-treated mice (A–C) After ten days of S.S or CpG-ODN+IFA-treatment, myeloid cells were isolated from the spleen of young and aged mice and co-cultured with naïve T cells isolated from young mice stimulated with anti-CD3 plus anti-CD28. Cytokine neutralizing antibodies were added separately or together. **(A)** After 24 h of co-culture, cells were collected and stained with anti-CD11b and anti-Gr1 antibodies, fixed, permeabilized and stained for arginase-1. Results represent the expression of arginase-1 on CD11b^+^Gr1^+^ gated cells. **(B)** After 48 h of co-culture, cells were lysed and prepared for arginase activity analysis. Results are expressed as mU of enzyme activity per mg of protein lysate. **(C)** Anti-mouse IL6 neutralizing antibody was added and, after 30 min culture, cells were collected, fixed, permeabilized, and stained with anti-CD11b, anti-Gr1, and anti-pSTAT3. pSTAT3 levels were analyzed on CD11b^+^Gr1^+^ cells. Results represent the expression of pSTAT3 (MFI) at 30 min by subtracting that measured at zero time (ΔMFI). (A–C) Data are pooled from two independent experiments (mean ± SEM; *n* = 3mice/group). *p < 0.05; **p < 0.01; ***p < 0.001.

To address the potential of IL-6 and IL-4 in the induction of arginase-1 expression in myeloid cells from CpG-ODN+IFA-treated mice, we added neutralizing antibodies against IL-6, IL-4, or both together, to co-cultures. When neutralizing antibody against IL-4 or IL-6 was separately added to the culture, arginase-1 expression in myeloid cells was substantially reduced at both ages (Figure 6A and Supplementary Figure 3). Unlike, when both neutralizing antibodies were present in the culture, the expression of arginase-1 in the case of myeloid cells from young mice was even more reduced (Figure 6A). The neutralization of IL-4, IL-6 or both cytokines in the cultures of myeloid cells from aged treated mice reduced arginase-1 expression to lower levels than in their saline solution counterparts.

We next analyzed whether IL-4 and/or IL-6 neutralization also affected arginase activity in myeloid cells. The addition of neutralizing antibody against IL-4 or IL-6 alone substantially reduced arginase activity in cultures of myeloid cells from young treated mice, but only when both antibodies were added together was arginase activity completely inhibited (Figure 6B). In cultures of myeloid cells from aged mice, the addition of neutralizing antibody against IL-4 or IL-6 alone was sufficient to completely abrogate arginase activity (Figure 6B). As expected, the same occurred when both antibodies were added together.

As it has been well established that IL-4/STAT6 pathway is involved in arginase induction in myeloid cells, we focused our studies on the participation of the IL-6/STAT3 phosphorylation pathway. Interestingly, when neutralizing antibody against IL-6 was added to the cultures, we observed a reduction in STAT3 phosphorylation levels in myeloid cells from young and aged CpG-ODN+IFA-treated mice (Figure 6C and Supplementary Figure 4). We also found that the levels of IL-10, another cytokine that may be responsible for STAT3 activation, were significantly reduced in cultures of T cells with myeloid cells from young or aged CpG-ODN+IFA-treated mice compared to their saline solution counterparts (data not shown). These results demonstrate that arginase activity and expression in myeloid cells from CpG-ODN+IFA-treated mice depend on IL-4 and IL-6 present in the T cell-myeloid cell extracellular microenvironment. Myeloid cells from aged CpG-ODN+IFA-treated mice require the presence of both cytokines for arginase induction, while IL-4 or IL-6 alone induces arginase in myeloid cells from young CpG-ODN+IFA-treated mice.

## DISCUSSION

We have found, in agreement with other authors [17–18], that lymphoid tissues of aged mice harbor increased numbers of myeloid cells CD11b^+^Gr1^+^. We have also demonstrated that these myeloid cells are more resistant to spontaneous apoptosis than their younger counterparts. Besides the well established evidence showing a myeloid-based output of the hematopoietic system with increasing age [11, 23–24], our results suggest the accumulation of myeloid cells in aged mice is likely to be related to an abnormally prolonged lifespan.

CpG-ODN were thought to be predominantly proinflammatory molecules. We and others have demonstrated the ability of CpG-ODN to act in aged mice as a strong adjuvant, resulting in the induction of a powerful antigen-specific immune response [12–14, 25]. However, current studies have shown the existence of CpG-ODN counter-regulatory mechanisms that prevent pathological immune-mediated damage [26–30]. Also, we have previously shown that CpG-ODN combined with IFN-γ is able to stimulate arginase, an anti-inflammatory enzyme, in murine macrophages [31].

Besides this, in previous work we have shown that CpG-ODN+IFA induces in young mice the expansion of a myeloid cell population with high arginase-1 expression and enzymatic activity, responsible for reduced T-cell proliferation and CD3z chain downregulation in the TCR complex in T-cells [15].

Here we describe for the first time that aged mice present an expansion of a myeloid cell population with immunoregulatory functions, compatible with MDSCs, after CpG-ODN+IFA treatment. Interestingly, in young mice, after reaching a peak on day 10 after treatment, myeloid cell numbers and their regulatory function diminished, returning to normal levels before day 25. However in aged animals, the myeloid cell population expanded by day 10 after treatment was maintained or even increased at least until day 40, conserving their suppressor function. This suggests that the inflammatory stimulus of CpG-ODN+IFA may expand MDSCs in spleen of aged mice for a longer period of time than in young mice, and preserve their regulatory function, probably as a way to prevent pathological inflammatory damage. As evidence of inflammatory response we found elevated levels of IL-6 in plasma of aged but not in young mice after CpG-ODN+IFA treatment (data not shown). Studies in aged mice and humans indicate that older adults have elevated levels of pro-inflammatory cytokines, clotting factors and acute phase reactants in the steady state, a phenomenon referred to as ‘inflamm-ageing’ [11, 32]. Our results showing that CD11b^+^Gr1^+^ myeloid cells from aged mice are more resistant to spontaneous apoptosis may suggest that the persistent expansion of these cells is due to heir prolonged lifespan. Chornoguz et al. suggest that inflammation protects MDSCs against extrinsic-induced apoptosis, resulting in MDSCs with a longer *in vivo* half-life [33] and similar results were observed by Ko et al. [34] in tumor-bearing mice.

A recent publication from Shirota et al. showed that intratumoral administration of CpG-ODN reduces the immune suppressive activity and number of monocytic MDSCs in a model of CT26 tumor-bearing mice [35]. Their study is based in a model of large, established tumors, where the number and immunosuppressive activity of MDSC was enhanced. The route of CpG-ODN administration also critically affects outcome, in that local but not systemic delivery of CpG-ODN altered the tumor microenvironment and was key to reducing the number and suppressive activity of MDSC. Zoglmeier et al. showed that CpG-ODN injection in tumor-bearing mice induces the expansion of CD11b^+^Gr1^+^ cells but does not increase their suppressive function [36]. They administrated CpG-ODN alone, whereas in our model CpG-ODN is combined with the slow-release delivery system, IFA, which could be one of the reasons for the differences observed in our study.

Among other factors, the age-related decrease of immune function is explained by an impaired T cell proliferative response [37–38]. Thus, our results show that splenocytes from aged saline solution-treated mice presented reduced proliferation levels, and, after CpG-ODN+IFA treatment, they showed even lower proliferative response. When myeloid cells isolated from spleen of aged-treated mice were cultured with T-cells from young animals, they suppressed their proliferation. On a per cell basis, myeloid cells from aged treated mice present the same ability to suppress T-cell proliferative response as those from their younger counterparts. The suppression of T-cell proliferative response exerted by splenic MDSCs from aged CpG-ODN+IFA-treated mice is mediated by arginase activity, as the addition of the arginase inhibitor, nor-NOHA, to the cultures almost completely restored T-cell proliferation. These results correlate with the increased L-arginine uptake in myeloid cells from young CpG-ODN+IFA-treated mice, resulting in the depletion of this amino acid from the cell microenvironment [15]. However, we cannot discard other mechanisms activated in these cells that could, to a lesser extent, also be responsible for the suppression.

Many reports established that factors and signals present in MDSCs microenvironment may induce their activation, up-regulating different suppressor mechanisms. These factors are produced mainly by activated T-cells, tumor stromal cells or induced by different bacterial and viral products [1, 8]. Several studies have indicated that the signaling pathway downstream of IL-4Rα (activated by the binding of either IL-4 or IL-13) and STAT6 plays an important role in MDSC activation [1, 39–40]. In addition, Qualls et al. demonstrated that STAT3-activating cytokines IL-6, IL-10, and G-CSF, produced by mycobacteria-infected macrophages, are critical for arginase-1 expression [20]. Similarly, many studies have shown that STAT3 is one of the main transcription factors that regulate the expansion of MDSCs [1, 41–42], but little is known about the role of IL-6 and STAT3 in MDSCs activation. We observed increased phosphorylation levels of STAT3 and STAT6 in young and aged myeloid cells from CpG-ODN+IFA-treated mice. Augmented STAT3 and STAT6 phosphorylation in MDSCs from CpG-ODN+IFA-treated mice correlated with their increased arginase activity and arginase-1 expression at both ages.

No significant difference was found in IL-4 levels in cultures of myeloid cells isolated from young or aged mice after CpG-ODN+IFA-treatment compared to their saline solution counterparts. However, myeloid cells from CpG-ODN+IFA-treated mice presented augmented STAT6 phosphorylation, correlating with higher expression of CD124 (IL-4Rα), which could be involved in the elevated response of these cells to IL-4 resulting in arginase induction. Similarly, a recent report demonstrated that IL-6, acting on a cell-autonomous level, directly induced the expression of IL-4Rα, and therefore primed macrophages for IL-4 dependent activation of STAT6 [43]. Interestingly, we found elevated levels of IL-6 in cultures of myeloid cells from CpG-ODN+IFA-treated mice along with augmented STAT3 activation. Our observations that the neutralization of each IL-4 or IL-6 in the culture medium of myeloid cells from aged CpG-ODN+IFA-treated mice lead to a reduction of arginase-1 expression and enzyme activity, indicate that the presence of both cytokines together is required for arginase induction in these cells, whereas in myeloid cells from young treated mice, the presence of IL-4 or IL-6 alone is sufficient to induce arginase-1 expression and activity. IL-4 and IL-6 may be synergizing for arginase induction in myeloid cells from young treated mice, as arginase activity was completely reduced when both cytokines were neutralized.

Several cellular signaling pathways involved in eliciting immune responses have also been shown to be defective in different cell types during aging [17, 44–46]. Therefore, the requirement of both IL-4/STAT6 and IL-6/STAT3 pathways to elicit arginase induction in myeloid cells from aged CpG-ODN+IFA mice, unlike the case of their younger counterparts, might be linked to age-associated changes in signal transduction functions, although further studies are required to test this hypothesis.

In summary, this work adds more information to the growing evidence suggesting that the expansion of myeloid cells with suppressor function may represent a common response to different forms of inflammation. Aged mice harbor elevated numbers of myeloid cells, possibly in response to their basal inflammatory conditions which after an inflammatory stimulus such as CpG-ODN+IFA, expand for longer time than in young mice and upregulate their suppressive abilities. These cells might be contributing to the dysregulation of the immune system during aging, to the inability to swiftly combat infection and to an increased susceptibility to chronic disease states and autoimmune conditions.

## METHODS

### Synthetic oligodeoxynucleotides

The synthetic oligodeoxynucleotide used was CpG-ODN 1826; B-class oligodeoxynucleotide (5′-TC CATGACGTTCCTGACGTT-3′) synthesized with a nuclease-resistant phosphorothioate backbone and containing no LPS contaminants (Operon Technologies., Alameda, CA, USA). CpG-ODN stock solution was prepared in sterile apyrogenic 0.9% NaCl saline solution (B. Braun Medical S.A, Mar del Plata, Buenos Aires, Argentina). The endotoxin content in oligodeoxynucleotide after reconstitution (1mg/mL), determined by a standard Limulus amebocyte lysate assay (BioWhittaker Inc., Walkersville, MD, USA), was < 1 endotoxin U/mL.

### Laboratory animals and treatments

Six to 8-week-old (young) female BALB/c mice were provided by the Veterinary School of the National University of La Plata animal production area (La Plata, Argentina). 18–20-month-old (aged) female mice were received as 6- to 8-weeks old and maintained in our animal facility until use according to the terms of the *Guide to the Care and Use of Experimental Animals*, published by the Canadian Council on Animal Care (with the assurance number A5802-01 assigned by the Office of Laboratory Animal Welfare (NIH)). Our Institutional Experimentation Animal Committee (authorization No. 15-01-44195 and HCD resolution 450/07) also approved the animal handling and experimental procedures. CpG-ODN, at 15.7 nmol/mouse, emulsified in IFA (Sigma-Aldrich, St. Louis, MO), was injected subcutaneously (s.c) into 2 sites of each mouse (0.2 mL/site). Control mice received injections of saline solution (S.S) alone. Splenocyte suspensions were obtained 10 days or, when indicated, 25, 40 and 80 days after treatment.

### Spleen cells culture

Spleens were surgically removed and red blood cells were lysed using RBC lysing buffer (Sigma-Aldrich). After washing, cell suspensions were cultured in GIBCO^®^ RPMI 1640 medium (Life Technologies, Argentina) supplemented with 10% heat-inactivated fetal bovine serum (PAA Laboratories GmbH, Linz, Austria), 2 mM GIBCO^®^ Glutamax, 100 U/mL Penicillin and 100 μg/mL Streptomycin (all from Life Technologies) and 50 μM 2-mercaptoethanol (Sigma-Aldrich) in a humidified incubator at 37°C and 5% CO_2_ for the period of time specified for each experiment.

When indicated, neutralizing antibodies were added at the beginning of the cultures: NA/LE anti-mouse IL4 (11B11) at a final concentration of 1 μg/mL, NA/LE anti-mouse IL6 (MP5-20F3) 1 μg/mL, NA/LE anti-mouse IL10 (JES5-2A5) 1 μg/mL, and anti-mouse isotype-matched control antibodies were obtained from Becton Dickinson Argentina or eBioscience (San Diego, CA, USA). Cytokine neutralization was controlled by measurement of their respective levels by ELISA in culture supernatants.

### Flow cytometry analysis

Cells were pre-incubated with anti-CD16/32 (2.4G2) for 15 min at 4°C, and then stained with fluorochrome-labeled antibodies for 30 min at 4°C. Cells were washed twice and 7-AAD was then added to exclude dead cells. The antibodies used against mouse CD11b (M1/70), Gr1 (RB6-8C5), Ly6G (1A8), Ly6C (AL-21), CD31 (390), CD124 (mIL4R-M1), CD86 (GL1), MHC class II (2G9), CD274 (PD-L1, clone MIH5), CD273 (PD-L2, clone TY25) were purchased from Becton Dickinson Argentina. For intracellular staining of arginase-1, cells were first stained with fluorochrome-labeled antibodies against mouse CD11b and Gr1. Then cells were permeabilized with BD Cytofix/Cytoperm™ Plus kit (Becton Dickinson Argentina), following the manufacturer's protocols, before being incubated for intracellular arginase-1 (8C9) (Santa Cruz Biotechnology, Santa Cruz, CA, USA) or isotype-matched control antibody (Becton Dickinson Argentina).

For the apoptosis assay, mitochondrial depolarization was measured using 50 nM tetramethylrhodamine ethyl ester (TMRE; Invitrogen) [47]. Cells were stained with fluorochrome-labeled antibodies against mouse CD11b and Gr1 for 30 min at 4°C and then incubated with TMRE for 30 min at 37°C. Cells incubated with 100 μM FCCP (carbonyl cyanide 4-(trifluoromethoxy)phenylhydrazone) were used as positive controls. Data acquisition was performed on a FACS Canto II cytometer (BD Biosciences) and analyzed using FlowJo software (Tree Star Inc., Ashland, OR).

### Arginase activity assay

Cells were lysed with 0.1% Tritón X-100 (Sigma-Aldrich) plus protease inhibitors (Sigma-Aldrich) for 30 min. Arginase activity was measured in cell lysates by colorimetric assay for the detection of urea, as described by Corraliza et al. [48] with slight modifications [31]. One unit of enzyme activity was defined as the amount of enzyme that catalyzed the formation of 1 μmol of urea/min.

### Cytokine detection assay

Concentrations of different cytokines were measured by standard sandwich ELISA following instructions from the manufacturer and standardized with recombinant murine cytokines. The antibody pairs used were as follows (listed by capture/biotinylated detection): IL-6, MP5-20F3/MP5-32C11; IL-4, 11B11/BVD6-24G2; IL-10, JES5-2A5/JES5-16E3. All antibodies were obtained from Becton Dickinson Argentina or eBioscience (San Diego, CA, USA). The concentrations were expressed in relation to standard curves constructed by assaying serial dilutions of the respective murine standard cytokine.

### Cell isolation

A single-cell suspension was prepared from the spleen of mice and washed in MACS buffer. CD11b^+^ cells were isolated using corresponding MACS magnetic microbeads (Miltenyi Biotec, Auburn, CA), and the purity of CD11b^+^Gr1^+^ cells was routinely > 95% as assessed by flow cytometry.

To purify T-cells, splenocytes from wild-type 6–8 week-old female mice were stained with APC-CD90.2 (53–2.1; Becton Dickinson Argentina) and isolated using cell sorting on a FACSAria II cell sorter (BD Biosciences). Purity was routinely > 98%.

### T-cell stimulation and *in vitro* suppression assays

For mitogenic-induced cell proliferation, splenocytes (2 × 10^6^cell/mL) were cultured for 72 h in round-bottomed 96-well plates in the presence or absence of ConA (5 μg/mL) (Sigma-Aldrich). 1 μCi [3H]-thymidine (Dupont NEN, Boston, MA, USA) was added to each well and, 18 h later, plates were harvested and then processed for measurement of incorporated radioactivity.

For proliferation measurement in co-cultures, CD11b^+^ cells were cultured in supplemented medium in triplicate in round-bottomed 96-well plates at 1 × 10^6^/mL. In parallel, 1 × 10^6^/mL splenic purified CD90^+^ T-cells from normal mice were stimulated with anti-CD3 (1 mg/mL) plus anti-CD28 (1 mg/mL) for 24 h. The stimulated T-cells were then cultured with the CD11b^+^ cells (at a 1:1 ratio) for an additional 48 h. To block arginase, 40 μM nor-NOHA inhibitor (N^W^-hydroxyl-nor-L-arginine) (Calbiochem, San Diego, CA, USA) was added at the beginning of culture. T-cell proliferation was tested by [3H]-thymidine incorporation in a liquid scintillation counter.

### Intracellular staining for STAT phosphorylation

After 30, 90, or 240 min of co-culture, cells were stained for pSTAT3 and pSTAT6 according to manufacturer's instructions (Becton Dickinson Argentina). Cells were first fixed with 2% paraformaldehyde for 10 min at 37°C and permeabilized with 90% ice-cold methanol for 30 min at 4°C. The cells were washed and stained with anti-Stat6 (pY641) or anti-Stat3 (pY705) antibodies (Becton Dickinson Argentina) for 30 minutes. Stimulated myeloid cells with IL-6 (100 ng/mL), or with IL-4 (10 ng/mL) were used as positive controls. Data acquisition was performed on a FACS Canto II Cytometer.

### Statistical analysis

Results are expressed as the mean ± SEM. Data were analyzed using GraphPad Prism software (GraphPad Software, San Diego, CA). Data analysis included the unpaired Student *t* test, one-way ANOVA and two-way ANOVA followed by a Bonferroni's post test. All data were considered statistically significant for *p* values < 0.05.

## SUPPLEMENTARY FIGURES


