# Supplementary material for: Expansion of myeloid-derived suppressor cells with arginase activity lasts longer in aged than in young mice after CpG-ODN plus IFA treatment
Source: Oncotarget. 2015 Apr 13;6(15):13448–61. doi: 10.18632/oncotarget.3626 (PMC4537026; doi:10.18632/oncotarget.3626)
Supplement: Supplementary file 1 [file oncotarget-06-13448-s001.pdf]

SUPPLEMENTAL FIGURES

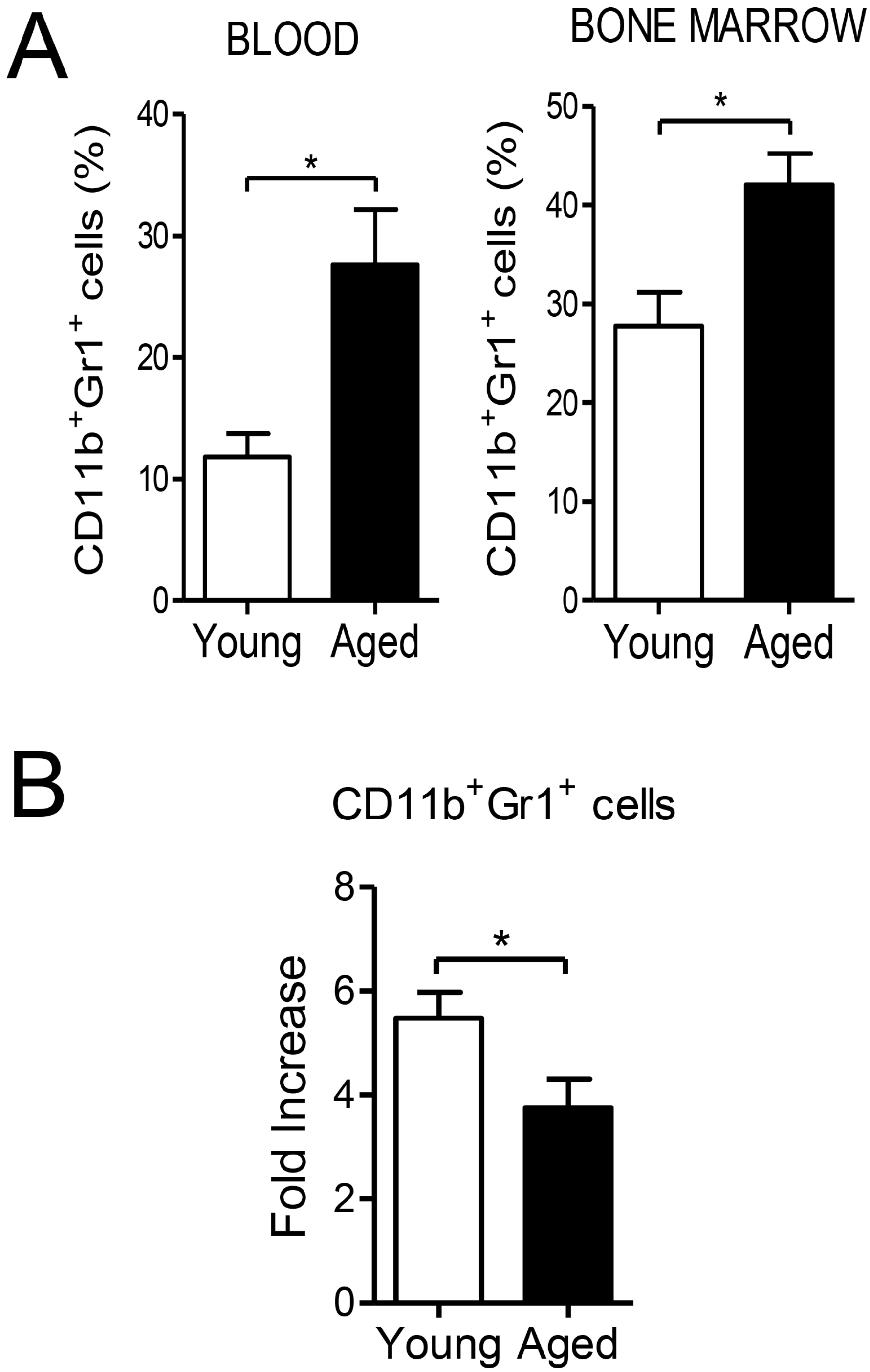

Supplementary Figure S1: Myeloid cells in spleen of aged mice. (A–B) Cells from blood and bone marrow compartments of aged mice were stained with anti-CD11b and anti-Gr1 antibodies and analyzed by flow cytometry. **(A)** Percentage of $CD11b^+Gr1^+$ cells are presented. **(B)** Splenocytes were collected from mice ten days after CpG-ODN+IFA or S.S treatment stained with anti-CD11b and anti-Gr1 and flow cytometry analysis was performed. Fold increase of $CD11b^+Gr1^+$ cells numbers from young and aged mice after CpG-ODN+IFA treatment related to their numbers in spleen of young or aged S.S-treated mice. Data are from three independent experiments; mean ± SEM ($n = 4$ mice/group) $*p < 0.05$.

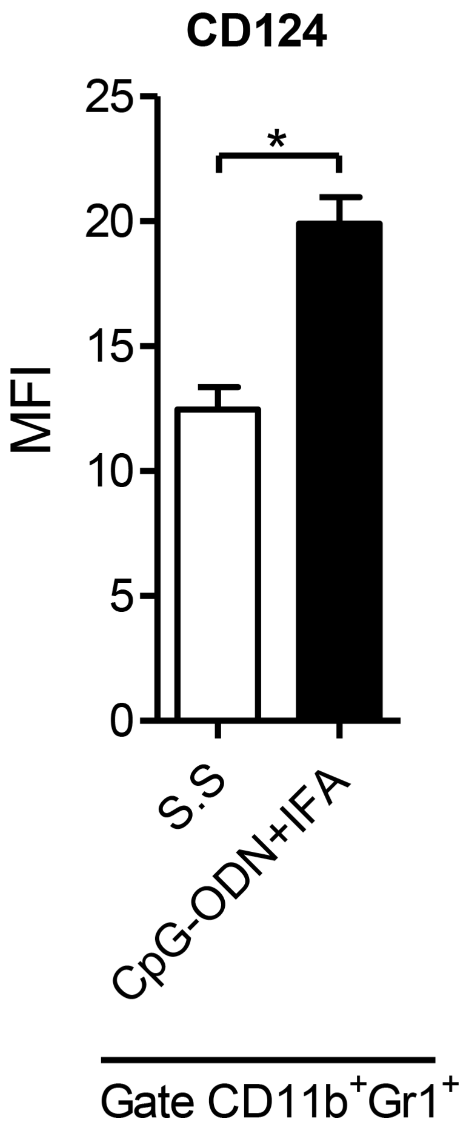

Supplementary Figure S2: CD124 expression in myeloid cells from young CpG-ODN+IFA-treated mice. Splenocytes were collected from young mice ten days after CpG-ODN+IFA or S.S treatment and flow cytometry analysis was performed. Mean Fluorescence Intensity (MFI) for CD124 on $CD11b^{+}Gr1^{+}$ gated cells are presented. Data are from three independent experiments; mean ± SEM ($n = 4$ mice/group) *$p < 0.05$; p values were calculated using t-test.

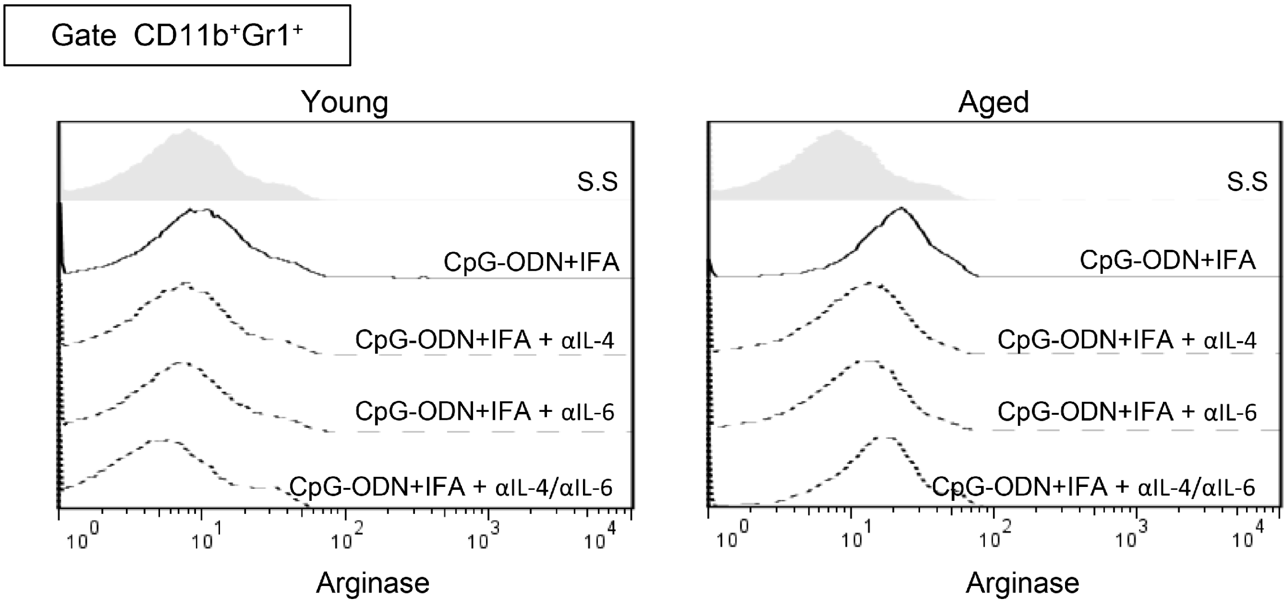

Supplementary Figure S3: Role of IL-4 and IL-6 in arginase induction in myeloid cells from CpG-ODN+IFA-treated mice. After ten days of S.S or CpG-ODN+IFA-treatment, myeloid CD11b$^+$ cells were isolated from the spleen of mice and were cultured with naïve T-cells isolated from young mice stimulated with anti-CD3 plus anti-CD28 Abs. Cytokine neutralizing antibodies were added at the beginning of the cultures: anti-mouse IL4 or/and anti-mouse IL6 After 24 h culture, cells were collected and stained with anti-CD11b and anti-Gr1 antibodies, fixed, permeabilized and then stained for arginase-1. Representative histograms of Arginase-1 expression in CD11b$^+$Gr1$^+$ gated cells are shown. Data are representative of one mice/age-group of one experiment of two performed.

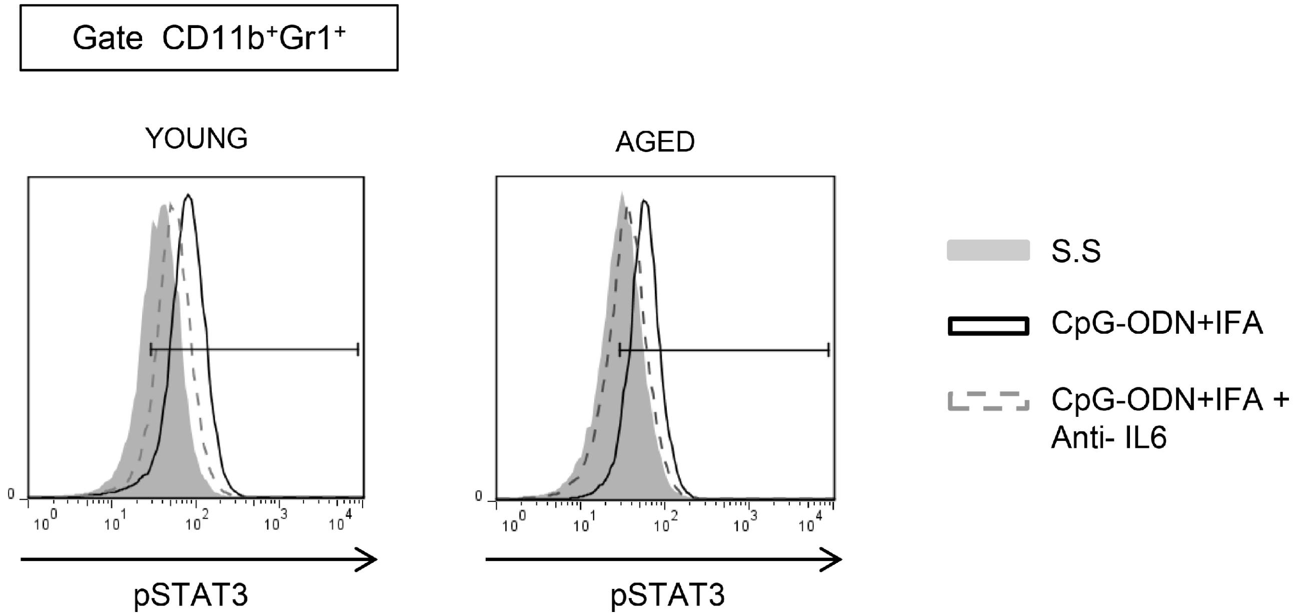

Supplementary Figure S4: pSTAT3 levels in myeloid cells from CpG-ODN+IFA-treated mice. After ten days of CpG-ODN+IFA-treatment, myeloid CD11b$^+$ cells were isolated from the spleen of young and aged mice and were cultured with naïve T-cells isolated from young mice stimulated with anti-CD3 plus anti-CD28 Abs. Anti-mouse IL6 neutralizing antibody was added at the beginning of the cultures in some wells and, after 30 min, cells were collected, fixed, permeabilized, and stained for anti-CD11b, anti-Gr1, and anti-pStat3. Representative histograms of pSTAT3 levels in CD11b$^+$Gr1$^+$ gated cells from young and aged mice are shown. Data are representative of one mice/age-group of one experiment of two performed.
